# Effects of Chronic Low-Temperature Exposure on the Behaviour of Adult Zebrafish (*Danio rerio*)

**DOI:** 10.3390/ani16182945

**Published:** 2026-09-19

**Authors:** Amalys Sofia Sanchez Garcia, Flavia Frabetti, Gabriella Tedeschi, Arianna Racca, Enrico Alleva, Mattia Toni

**Affiliations:** 1 Department of Biology and Biotechnologies “Charles Darwin”, Sapienza University, 00185 Rome, Italy; 2 Department of Medical and Surgical Sciences—DIMEC, University of Bologna, 40126 Bologna, Italy; flavia.frabetti@unibo.it; 3 Department of Veterinary Medicine and Animal Science (DIVAS), Università degli Studi di Milano, Via dell’Università 6, 26900 Lodi, Italy; gabriella.tedeschi@unimi.it; 4 Coordinating Research Centres (CRC) “Innovation for Well–Being and Environment” (I–WE), Università degli Studi di Milano, 20126 Milan, Italy; 5 Centre for Behavioural Sciences and Mental Health, Istituto Superiore di Sanità, Viale Regina Elena 299, 00161 Rome, Italy; arianna.racca@iss.it (A.R.); enrico.alleva@guest.iss.it (E.A.)

**Keywords:** zebrafish, *Danio rerio*, low temperature, chronic exposure, behaviour, exploration, anxiety-related behaviour, social preference

## Abstract

Temperature is a major environmental factor influencing fish behaviour and physiological function. In the present study, adult zebrafish were exposed to 18 °C for 21 days and compared with controls maintained at 26 °C to evaluate the behavioural effects of chronic low-temperature exposure. Behaviour was assessed using four individual-based paradigms—the novel tank diving test, light–dark preference test, social preference test, and mirror biting test—covering locomotion, exploration, social behaviour and anxiety-related responses. Chronic exposure to 18 °C was associated with a behavioural profile characterised by increased immobility, reduced exploratory activity, enhanced bottom-dwelling, and reduced active engagement with the mirror stimulus, whereas social preference towards conspecifics was largely preserved. Comparison with behavioural responses previously reported after 4 days of exposure to 18 °C suggests that several behavioural differences are still detectable after 21 days, indicating that the behavioural profile of fish maintained at 18 °C remained distinct from that of controls within the time window examined. These findings should be interpreted in light of the absence of independent tank-level replication. Previous studies conducted under comparable thermal conditions have reported molecular changes in the brain and in other neural-derived structures, such as the eye. Although these molecular findings were not generated in the present experiment and cannot be directly linked to individual behavioural endpoints, they provide a broader context for interpreting the behavioural effects of prolonged low-temperature exposure. Overall, the present findings show that chronic exposure to 18 °C is associated with persistent differences in locomotor, exploratory, and stimulus-directed behaviours, while social preference is comparatively preserved.

## Simple Summary

Temperature strongly affects how fish behave and function. In this study, adult zebrafish were kept for three weeks at a lower temperature (18 °C) and compared with fish maintained at a more favourable temperature (26 °C). Their behaviour was examined in different situations, including exploration of a novel environment, choice between light and dark zones, interaction with other conspecifics, and response to their own reflection. Fish exposed to cold water moved less, explored less, and spent more time inactive. They also showed a reduced active response to the mirror, while their tendency to stay near other fish remained largely unchanged. Comparison with previously published data obtained after acute exposure to the same temperature showed that several behavioural differences were still present after three weeks, indicating that prolonged exposure to cold was associated with a behavioural profile that remained distinct from that of control fish within the time window examined. Since each temperature condition was represented by a single home tank, these findings should be interpreted as individual-level observations within a non-replicated tank design. Previous studies under the same thermal conditions showed that cold exposure also changes proteins in the brain and in the eye, suggesting that temperature may affect both neural and sensory systems. Although this study was not designed to directly associate specific molecular changes with specific behaviours, the persistence of both molecular and behavioural alterations suggests a consistent biological response to cold. These findings show that temperatures compatible with survival can still markedly affect behaviour, with possible consequences for how fish cope with natural environments.

## 1. Introduction

Temperature is one of the main abiotic factors influencing the biology of ectothermic organisms and plays a crucial role in shaping fish physiology, behaviour, and ecological performance. It affects metabolism, growth, immune function, and ecological interactions, ultimately influencing organismal fitness [1,2,3].

As poikilotherms, fish depend on environmental temperature to determine body temperature and must therefore continuously adjust their physiology and behaviour in response to changing thermal conditions [4]. More broadly, temperature is a major determinant of fish performance, ecology, and biogeography, and its effects emerge through interactions among physiological, behavioural, and ecological processes [5,6,7].

Both increases and decreases in temperature can represent significant stressors. However, in the context of global climate change, the effects of warming have received far more attention than those of low-temperature exposure. Nevertheless, cold stress represents an ecologically relevant challenge, as it may arise from seasonal fluctuations, sudden drops in temperature, or anthropogenic disturbances [8,9].

The magnitude of the response depends not only on absolute temperature, but also on the degree of deviation from the thermal optimum, the rate of temperature change, and the duration of exposure [9,10,11]. Studies on cold-adapted fishes, including Arctic and Antarctic species, further illustrate how strongly temperature can constrain performance, activity, and acclimation capacity, with important consequences for survival and ecological persistence [12,13,14].

Exposure duration is particularly important since acute and chronic conditions may elicit distinct biological responses. Acute exposure generally triggers rapid responses aimed at restoring homeostasis, whereas more prolonged exposure may activate acclimation processes, namely more stable physiological and biochemical adjustments enabling the organism to better tolerate altered environmental conditions [7,10,11,15,16]. Behaviour represents a particularly sensitive endpoint of these processes, as it integrates the internal physiological state of the organism while providing an early indication of the impact of thermal stress.

Importantly, acclimation does not necessarily imply a full return to the original condition, but may instead involve the establishment of a new functional equilibrium at molecular, physiological, and behavioural levels [7,17].

The zebrafish (*Danio rerio*) is a particularly suitable model for investigating thermal stress because of both its broad thermal tolerance under natural conditions and its ecological relevance. The species has an approximate viability range of 6–43 °C, with tolerated temperatures ranging from about 4.5 to 42 °C [18,19]. In the wild, zebrafish inhabit South Asian environments characterised by marked daily and seasonal thermal fluctuations, including streams, ponds, and rice fields, where temperatures generally range from approximately 16.5 to 34 °C [20,21,22]. The thermal preference of the species lies around 26 °C, making this temperature an appropriate reference condition for controls [23].

Recent work comparing wild and laboratory zebrafish has further highlighted the usefulness of this model for thermal biology, showing that zebrafish retain substantial thermal plasticity and that thermal tolerance and preferred temperature are closely linked to environmental and physiological context [24,25,26].

Previous work, including studies from our laboratory, has shown that thermal manipulation in adult zebrafish induces coordinated responses at multiple levels of biological organisation, from molecular remodelling to altered behavioural output. In particular, proteomic analyses revealed that both low and high temperatures induce marked changes in brain protein expression under acute and chronic conditions [27,28,29,30], and chronic exposure to both 18 °C and 34 °C also alters the brain lipidome [31]. More recently, chronic exposure to the same non-optimal temperatures was also shown to affect the eye proteome in adult zebrafish, indicating that thermal effects are not restricted to the brain but extend to other neural-derived structures involved in sensory processing [32].

At the behavioural level, individual-based assays, including the novel tank diving test (NTT), light–dark preference test (LDT), social preference test (SPT), and mirror biting test (MBT), showed that acute exposure to 18 °C is associated with reduced locomotion, altered exploratory behaviour, and several behavioural changes commonly interpreted as anxiety-like responses, whereas acute exposure to 34 °C produces a partly opposite behavioural profile, characterised by lower anxiety-like responses and greater exploratory or approach-related activity [29].

Comparable behavioural data are also available for chronic high-temperature exposure, indicating that the behavioural effects observed acutely at 34 °C persist and become more pronounced after 21 days [33].

By contrast, although molecular data were already available for prolonged exposure to 18 °C [30], a systematic characterisation of the corresponding behavioural responses has so far been lacking.

The aim of the present study was therefore to evaluate the effects of chronic exposure to 18 °C for 21 days on the behaviour of adult zebrafish. To ensure direct comparability with the previously obtained acute data, we used the same experimental design and the same behavioural paradigms as those employed in the 4-day treatment based on NTT, LDT, SPT and MBT [29].

This approach enabled us to determine whether the behavioural alterations previously observed after acute exposure changed during prolonged exposure or remained evident after 21 days.

## 2. Materials and Methods

### 2.1. Ethical Statement

All animal procedures were carried out in accordance with the guidelines approved by the institutional Animal Care Committee and were authorised by the Italian Ministry of Health (protocol no. 290/2017–PR). All animal procedures were carried out in accordance with Directive 2010/63/EU, as amended by Regulation (EU) 2024/1262, and with Italian animal welfare legislation (Legislative Decree No. 26/2014, as recently amended by Decree-Law No. 183/2025). Fish health status and welfare were monitored daily throughout the thermal treatment and subsequent behavioural testing. No formal humane endpoints were reached during the study, and none of the procedures caused significant pain, lasting harm, or mortality.

### 2.2. Animals

A total of 40 experimentally naïve adult wild-type zebrafish (*Danio rerio*; 12 months old; approximately 1:1 male:female ratio) were used as focal experimental subjects.

Fish were selected to obtain an approximately balanced male:female ratio at the group level. However, sex was not recorded at the individual level during behavioural testing, and the exact number of males and females allocated to each temperature condition was therefore not available. Consequently, sex was not included as a factor in the behavioural analyses and potential sex-dependent behavioural responses to chronic low-temperature exposure could not be evaluated in the present study.

In addition, three adult zebrafish of comparable age and size were used exclusively as stimulus fish in the social preference test. These stimulus fish were not included in the behavioural analyses and were never used as focal subjects. Focal fish originated from a stock colony initially obtained from commercial suppliers and maintained in the zebrafish facility of the University of Bologna. Before the beginning of the experiment, animals were housed under standard conditions at 26 ± 1 °C.

Fish were randomly allocated to two home tanks (W 40 × D 30 × H 30 cm), with 20 fish per tank, at a stocking density of approximately 1 fish L^−1^. Group allocation was performed using a random number generator. Before thermal treatment, fish were allowed to acclimate to the experimental tanks for 10 days at 26 ± 1 °C.

No a priori exclusion criteria were established, and no animals or data points were excluded from the analyses.

### 2.3. Experimental Design Considerations

The present study was intentionally designed to match the housing, thermal-treatment and behavioural-testing procedures adopted in our previous work as closely as possible, in order to maximise comparability between acute and chronic exposure conditions [28,29,30,33]. In accordance with the principles of reduction and refinement, and given the social nature of zebrafish, subjects were maintained in groups rather than individually. For this reason, each temperature condition was represented by a single home tank, containing 20 fish.

The two home tanks were maintained in the same experimental room, under identical photoperiods, lighting, handling conditions, and equipment configurations. Assignment of temperature conditions to tanks was random. Temperature was continuously regulated using digital thermostats and independently monitored using both digital and analogue thermometers in order to minimise unwanted variability between tanks.

Because temperature was manipulated at the level of the home tank, the tank represented the experimental unit for thermal exposure, whereas individual fish represented observational units for behavioural assessment. Consequently, independent tank-level replication was not available in the present design, and potential tank-specific effects cannot be fully disentangled from the effects of temperature. For this reason, the behavioural comparisons reported here should be interpreted as individual-level observational comparisons between fish maintained under two highly standardised thermal conditions, rather than as fully replicated tank-level tests of temperature effects.

This tank-based design was chosen to ensure direct comparability with previous studies from our laboratory [28,29,30,33] and to comply with welfare and experimental-animal reduction criteria. However, we acknowledge that the absence of independent tank-level replication limits the strength of inference regarding temperature effects. This aspect should therefore be considered when interpreting the data. Future studies using multiple independent replicate tanks per temperature condition will be required to confirm the generality of the observed behavioural differences and to allow tank-level effects to be appropriately modelled.

### 2.4. Thermal Treatment

To initiate the low-temperature treatment, water temperature was gradually reduced from 26 °C to 18 °C at an approximate rate of 0.5 °C h^−1^. This was achieved by decreasing the thermostat setting by 1 °C every 2 h, allowing progressive thermal equilibration of the entire tank volume. Because thermal equilibration required time, the actual change in water temperature occurred gradually and corresponded to approximately 0.5 °C h^−1^. The cooling protocol was intentionally matched to that previously used in our acute low-temperature study [29] in order to ensure direct comparability between acute and chronic exposure conditions. No mortality or obvious signs of severe distress were observed during the temperature transition. Fish assigned to the experimental group were maintained at 18 ± 1 °C for 21 days, whereas control fish remained at 26 ± 1 °C for the same period.

### 2.5. Fish Husbandry During Acclimation and Thermal Exposure

Animal husbandry followed the same protocol previously used in our laboratory and described in earlier studies [29,30]. All procedures were performed in the same experimental room and using the same type of tanks and environmental conditions adopted in previous work [29,30].

Fish were maintained under a 13 h light/11 h dark photoperiod (lights on from 08:00 to 21:00). The internal arrangement of each home tank, including the heating coil, filter inlet and outlet pipes, and aeration system, was reproduced as consistently as possible across the two tanks. These elements may also have provided a degree of environmental complexity; however, no additional environmental enrichment was used.

Water temperature was regulated by a TK 150 aquarium chiller (TECO S.r.l., Ravenna, Italy) and digital thermostats (Eden 430, Eden s.r.l., Vicenza, Italy) connected to a heating coil (Eden 415, 230 V, 50/60 Hz, 80 W, Eden s.r.l., Vicenza, Italy). Temperature was checked daily using an analogue thermometer and monitored continuously throughout the treatment period.

Each tank was supplied with a constant flow of filtered water (600 L h^−1^) through an external filtration system (Eden 511 h, Eden s.r.l., Vicenza, Italy) and was continuously aerated (7.20 mg O_2_ L^−1^) using an aquarium aerator (Sicce AIRlight, SICCE s.r.l, Vicenza, Italy, 3300 cc/min 200 L/h). Water conductivity ranged between 400 and 500 µS cm^−1^. The main physicochemical parameters of the water, including total ammonia (NH_3_/NH_4_^+^), nitrite (NO_2_^−^), pH, total hardness, carbonate hardness (dKH), phosphate (PO_4_^3−^), copper (Cu), and chlorine (Cl_2_), were checked at least twice weekly using a Sera Aqua-test Box Kit (Sera Italia srl, Bologna, Italy) and eSHa Aqua Quick Test strips (eSHa Lab, Maastricht, The Netherlands). No relevant differences were detected between the control and treatment tanks.

Fish were fed three times daily (10:00, 14:00 and 18:00) with a commercial dry granular diet (TropiGranMIX, Dajana per sro, Bohuňovice, Czech Republic). Food was provided in a way that allowed fish at the two temperatures to feed according to their appetite. During both the acclimation period and the thermal treatment, fish behaviour was inspected daily in order to monitor welfare-related indicators, including abnormal swimming, loss of equilibrium, impaired feeding, and other signs of distress.

### 2.6. Behavioural Tests: General Design

Behaviour was assessed using four established paradigms: the novel tank diving test (NTT), light–dark preference test (LDT), social preference test (SPT), and mirror biting test (MBT). These individual-based assays are widely used in adult zebrafish and rely on spontaneous behavioural tendencies, allowing the detection of treatment-induced changes across complementary domains. The NTT and LDT primarily probe exploratory and anxiety-like responses and are based on innate tendencies such as vertical distribution in a novel environment and preference for dark over brightly illuminated zones [34]. In particular, the NTT, often considered the zebrafish counterpart of the rodent open field/novelty paradigm, exploits the characteristic diving response displayed by fish when introduced into an unfamiliar tank, whereas the LDT is based on adult zebrafish scototaxis. The SPT and MBT were used to investigate social and stimulus-directed behaviour [35]. The SPT takes advantage of the strong tendency of zebrafish to approach and remain near conspecifics and measures the social preference of an individual fish towards conspecifics confined in a separate adjacent tank. In contrast to shoaling assays, it does not assess group organisation or shoal cohesion. The MBT assesses the response evoked by mirror-image stimulation, which may include approach, boldness-related behaviour, and aggressive acts such as biting [36,37,38]. Taken together, this battery of tests provides an integrated behavioural profile spanning locomotor activity, exploration, anxiety-like behaviour, social preference, and active engagement with a salient species-relevant social-like stimulus. To maximise comparability with our previous studies, all behavioural tests were performed using the same apparatus and general procedures previously adopted in our laboratory [29,33]. Sample size was determined on the basis of published evidence indicating that robust behavioural effects in zebrafish may be detected with *n* = 12–15 fish per group [39], and was increased to 20 fish per group in the present study in order to obtain more robust and fine-grained statistical estimates while remaining consistent with reduction principles and group-housing requirements.

No single primary outcome measure was predefined, as the study was designed to characterise the behavioural profile induced by chronic low-temperature exposure across multiple validated behavioural paradigms.

The water temperature in all behavioural apparatus matched that of the home tank in which the fish had been housed and was maintained within ±1 °C throughout testing. To avoid disturbing the animals, the test apparatus was not aerated during behavioural assessment. Before testing, each fish was gently transferred using a beaker to an individual waiting tank (W 15 × D 10 × H 10 cm) and left undisturbed for 30 min. Following this acclimation period, each fish underwent the entire behavioural battery on the same day.

To minimise potential interference between consecutive tests [36,40], fish were randomly assigned to one of four counterbalanced test sequences, in which NTT and LDT alternated as the first or second assay and SPT and MBT alternated as the third or fourth assay. The 30 min acclimation period was performed only once, before the first behavioural assay.

At the end of each behavioural session, water was discarded, and the apparatus was rinsed and refilled with clean water. Behavioural tests were conducted under diffuse room lighting to avoid directional illumination that might bias fish responses.

Each test lasted 10 min and was video-recorded using a Logitech C170 webcam (Logitech Europe S.A., Lausanne, Switzerland), positioned 1 m above the apparatus in the LDT, SPT and MBT, and 1 m in front of the apparatus in the NTT.

Behavioural testing was conducted during the light phase between 10:00 and 17:00.

All behavioural measures were recorded and analysed at the level of the individual fish, allowing subject-specific data to be retained across all behavioural paradigms.

Testing of the entire cohort required approximately one day per treatment group. Therefore, fish exposed to 18 °C were assessed immediately at the end of the 21-day exposure period, whereas control fish were tested on the following day. All other testing conditions were kept identical between the two testing days. This approach ensured a uniform duration of thermal exposure within each treatment group.

### 2.7. Novel Tank Diving Test (NTT)

The NTT was used to assess locomotor activity, vertical exploration, and anxiety-like behaviour. The apparatus consisted of a transparent glass tank with a lower triangular base [41], with 30 cm sides, filled with 4.7 L of water. The external walls were covered with white paper to minimise reflections.

For the analysis of vertical exploratory behaviour, the tank was virtually divided into three equal horizontal zones (bottom, middle, and top). The parameters analysed were immobility time, number of immobility episodes, number of transitions to the bottom, middle, and top zones, and time spent in each zone.

### 2.8. Light–Dark Preference Test (LDT)

The LDT was used to assess exploratory activity and anxiety-like behaviour. This paradigm is based on the natural preference of adult zebrafish for dark environments (scototaxis) and their relative avoidance of brightly illuminated zones [42]. The apparatus consisted of a rectangular plastic tank (L 33 × W 18 × H 18 cm) divided into two equal compartments. One half had black lateral walls and a black base, whereas the other half was made of opaque white plastic. The dark compartment was further shielded from ambient light by an opaque black lid [42]. The tank was filled with 4 L of water.

A central starting compartment (W 7 × D 18 × H 18 cm), half black and half white, was created by two transparent transverse septa and two sliding doors. After the fish had been placed in the central zone for 30 s, both doors were simultaneously lifted to allow free exploration of the two compartments. The parameters analysed were time spent in the dark zone and number of transitions between compartments.

### 2.9. Social Preference Test (SPT)

Adult zebrafish are highly social vertebrates [43] and display group-related behaviours such as shoaling and schooling [44,45]. The experimental setup for the SPT consisted of three rectangular tanks (W 28 × D 25 × H 16 cm) aligned side by side: an experimental tank in the centre, a conspecific tank on one side, and an empty tank on the other side. Each tank was filled with 4 L of water.

The focal fish was placed in the central tank, whereas the conspecific tank contained three adult zebrafish of comparable age and size, used exclusively as social stimulus fish. These stimulus fish had never previously been housed with the focal fish and were not used as focal subjects in any behavioural test. During the week preceding the tests, the stimulus fish were habituated to the conspecific tank for 3 h per day. The same three stimulus fish were used as the social stimulus throughout all SPT sessions.

These stimulus fish were maintained separately from the experimental subjects under standard housing conditions at 26 ± 1 °C and were not exposed to the chronic low-temperature treatment. During SPT testing, the stimulus fish remained in the conspecific tank at 26 ± 1 °C. Thus, the social stimulus was standardised across all trials, whereas only the focal fish differed in thermal history and testing temperature. Because the three compartments were physically separated and social interaction relied exclusively on visual stimuli, maintaining the stimulus fish under identical thermal conditions across all sessions allowed standardisation of the social cue presented to the focal fish. The position of the social stimulus (left or right side of the experimental tank) was counterbalanced across tests.

At the beginning of the test, two opaque black panels were placed between the tanks to prevent visual contact. After the focal fish had settled for 30 s, the panels were gently removed. The experimental tank was virtually divided into three zones, and the zone adjacent to the conspecific tank was defined as the social zone. The parameters analysed were immobility time, number of immobility episodes, time spent in the social zone, and number of transitions into the social zone.

### 2.10. Mirror Biting Test (MBT)

Mirror image stimulation is a well-established paradigm for assessing social approach and aggressive behaviour in zebrafish [35]. The apparatus consisted of a rectangular tank (W 28 × D 25 × H 16 cm) filled with 4 L of water and equipped with a mirror (D 16 × H 14 cm).

At the beginning of the test, the fish was introduced into the tank and allowed to settle for 30 s, after which the mirror was positioned at an angle of 22.5° along the long side of the tank [36,37,38]. The side on which the reflected image appeared (left or right) was counterbalanced across tests. The parameters analysed were immobility time, number of immobility episodes, absolute turn angle, number of entries into the mirror approach zone, time spent in the mirror approach zone, distance travelled within the mirror approach zone, and number of mirror bites.

### 2.11. Video Tracking and Statistical Analysis

Videos were analysed using a combination of direct observer scoring and automated tracking with ANY-maze^®^ software (version 7.65, Stoelting Co., Wood Dale, IL, USA). Manual scoring was used for behavioural endpoints that could be reliably identified by visual inspection, including immobility time, number of immobility episodes, transitions between zones, time spent in specific zones, entries into the social and mirror approach zones, and mirror biting events. This approach allowed direct verification of behavioural events and minimised potential artefacts arising from occasional tracking inaccuracies by the software when fish were located close to tank walls, reflective surfaces, or the mirror stimulus. Automated tracking was used for locomotor variables requiring continuous reconstruction of swimming trajectories, including absolute turn angle and distance travelled within the mirror approach zone.

Behavioural videos were analysed blind to treatment condition in order to minimise observer bias. Statistical analyses were performed using Origin 2018 (version 9.5.1.195, OriginLab, Northampton, MA, USA). To quantify individual-level behavioural differences between fish maintained under the two thermal conditions, unpaired two-tailed *t*-tests with Welch’s correction were used. For consistency, t statistics are reported for the contrast 18–26 °C; therefore, positive t values indicate higher values at 18 °C, whereas negative t values indicate lower values at 18 °C relative to 26 °C. Data are presented as mean ± SEM, and statistical significance was set at *p* ≤ 0.05. Exact statistical details for each behavioural comparison are reported in Supplementary Table S1.

Because thermal treatment was applied at the level of the home tank, whereas behavioural measurements were collected at the level of individual fish, the tank represented the experimental unit for thermal exposure and individual fish represented observational units for behavioural assessment. Each temperature condition was represented by a single home tank; therefore, independent tank-level replication was not available. Consequently, it was not possible to include tank as a random effect or to perform an independent tank-level statistical analysis. The behavioural comparisons reported here should therefore be interpreted as individual-level observational comparisons between fish maintained under two highly standardised thermal conditions, rather than as fully replicated tank-level tests of temperature effects. Accordingly, the resulting *p* values should be interpreted as descriptive statistics for individual-level differences within this design, not as evidence from a fully replicated tank-level analysis.

Sex was not included as an experimental factor because individual sex was not recorded during behavioural testing. Therefore, the analyses were not designed to evaluate sex-dependent effects or temperature-by-sex interactions. Given the tank-based design of the study, possible differences in sex composition between temperature conditions cannot be fully excluded as a source of behavioural variability. A replicated design with individual sex identification would be required to formally test sex effects and temperature-by-sex interactions while accounting for tank-level variance.

## 3. Results

Behavioural effects of chronic exposure to 18 °C were assessed across four individual-based paradigms evaluating complementary domains, including locomotion, exploratory activity, anxiety-related responses, social preference, and mirror-directed behaviour. The results obtained in each test are reported below, focusing on individual-level comparisons between fish maintained at 18 °C and control fish maintained at 26 °C.

### 3.1. Novel Tank Diving Test

**Figure 1:**
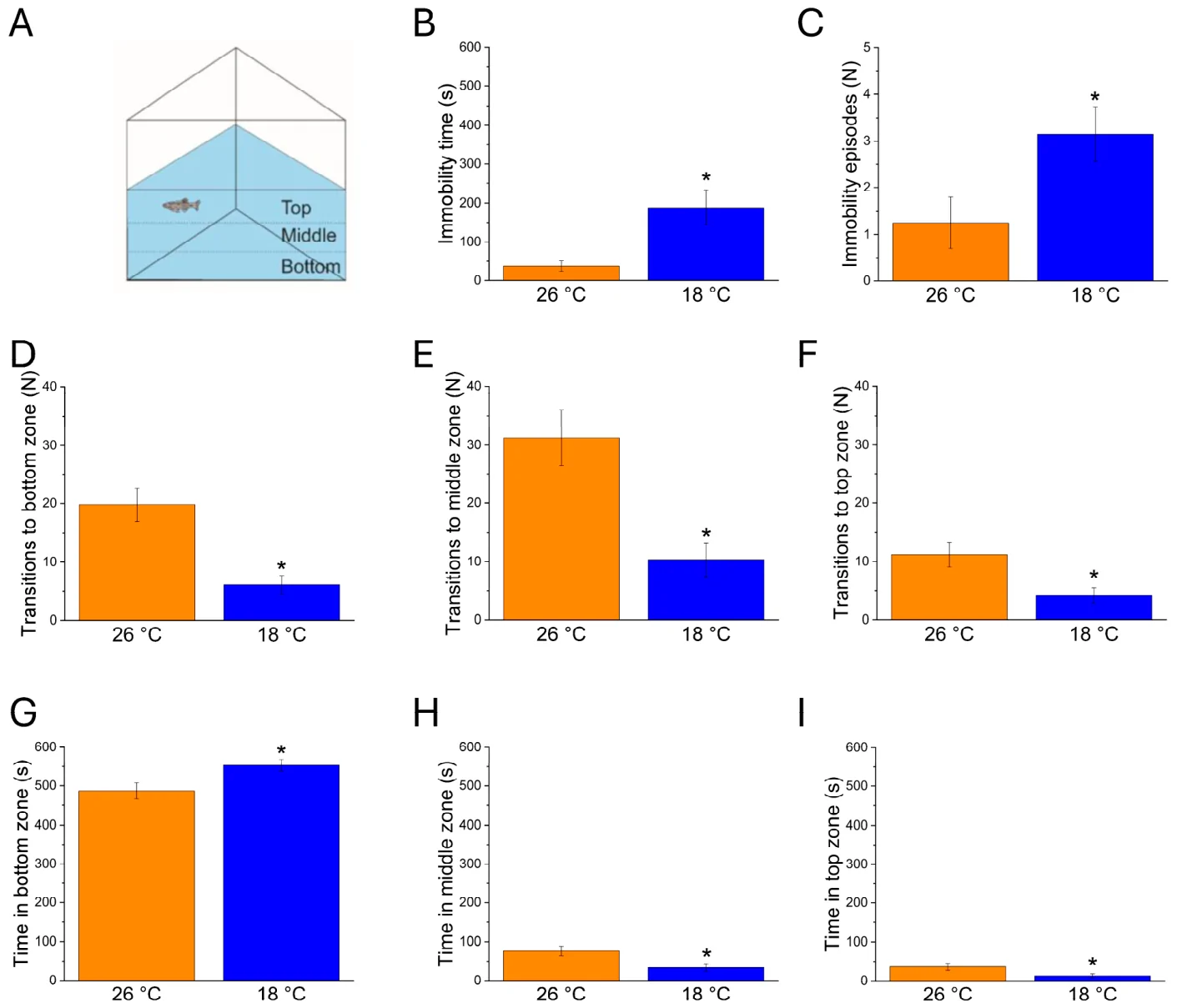
Novel tank diving behaviour following chronic exposure to low temperature. (**A**) Schematic representation of the apparatus and virtual division into bottom, middle and top zones. (**B**) Immobility time, (**C**) number of immobility episodes, (**D**) transitions to the bottom zone, (**E**) transitions to the middle zone, (**F**) transitions to the top zone, (**G**) time spent in the bottom zone, (**H**) time spent in the middle zone and (**I**) time spent in the top zone. Data are expressed as means ± SEM. Significant differences were assessed by unpaired two-tailed *t*-tests with Welch’s correction. * *p* ≤ 0.05 versus 26 °C. *N* = 20 per group.

In the NTT (Figure 1A), zebrafish exposed to 18 °C for 21 days showed a significant increase in both total immobility time (t(22.42) = 3.28, *p* = 0.0034) and number of immobility episodes (t(37.83) = 2.35, *p* = 0.0241) compared with control fish maintained at 26 °C (Figure 1B,C), indicating enhanced immobility and freezing-like behaviour under chronic low-temperature exposure.

Vertical exploratory behaviour was assessed by quantifying transitions between the bottom, middle, and top zones. Fish maintained at 18 °C displayed a significant reduction in the number of transitions to all three zones compared with controls (bottom: t(29.38) = −4.23, *p* = 0.0002; middle t(31.39) = −3.71, *p* = 0.0008; top: t(32.56) = −2.80, *p* = 0.0084; Figure 1D–F), consistent with reduced activity along the vertical axis.

Analysis of spatial distribution further showed that zebrafish exposed to 18 °C spent significantly more time in the bottom zone (t(34.98) = 2.59, *p* = 0.0140), together with a marked reduction in the time spent in the middle (t(36.80) = −2.66, *p* = 0.0114) and top zones (t(29.81) = −2.20, *p* = 0.0358; Figure 1G–I).

### 3.2. Light–Dark Preference Test

**Figure 2:**
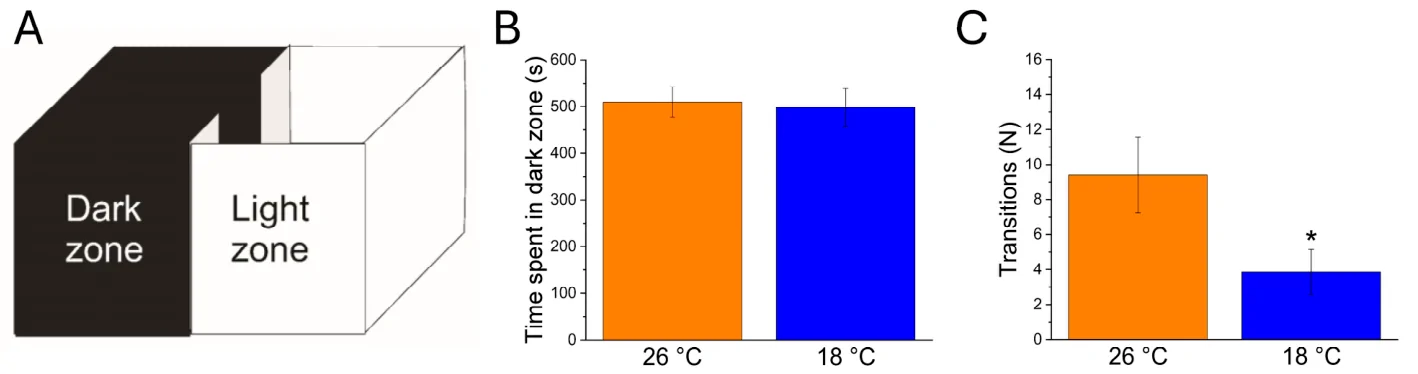
Light–dark preference behaviour following chronic exposure to low temperature. (**A**) Schematic representation of the apparatus. (**B**) Time spent in the dark zone and (**C**) number of transitions between the light and dark zones. Data are expressed as mean ± SEM. Significant differences were assessed by unpaired two-tailed *t*-tests with Welch’s correction. * *p* ≤ 0.05 versus 26 °C. *N* = 20 per group.

In the LDT (Figure 2A), zebrafish maintained at 18 °C did not differ significantly from control fish kept at 26 °C in the time spent in the dark zone (Figure 2B).

However, fish exposed to 18 °C showed a significant reduction in the number of transitions between the light and dark zones (t(30.85) = −2.20, *p* = 0.0354; Figure 2C).

### 3.3. Social Preference Test

**Figure 3:**
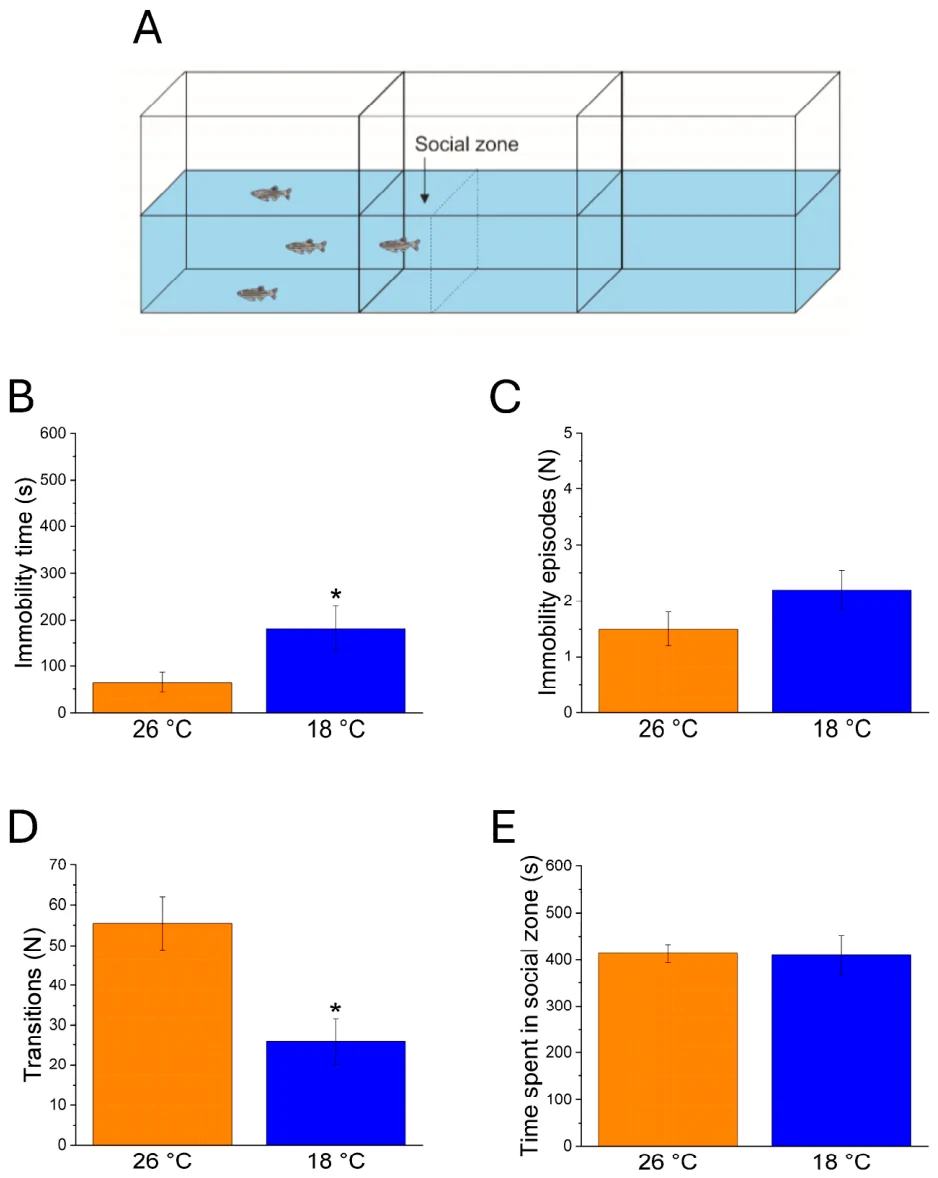
Social preference behaviour following chronic exposure to low temperature. (**A**) Schematic representation of the apparatus. Whole-tank analysis: (**B**) Immobility time and (**C**) number of immobility episodes. Social zone analysis: (**D**) Number of transitions between social and non-social zones and (**E**) time spent in the social zone. Data are expressed as mean ± SEM. Significant differences were assessed by unpaired two-tailed *t*-tests with Welch’s correction. * *p* ≤ 0.05 versus 26 °C. *N* = 20 per group.

In the SPT (Figure 3A), zebrafish exposed to 18 °C exhibited a significant increase in immobility time compared with controls (t(26.46) = 2.24, *p* = 0.0338), whereas the number of immobility episodes was not significantly altered (Figure 3B,C). In parallel, fish maintained at 18 °C showed a reduced number of transitions between the social and non-social zones (t(37.35) = −3.44, *p* = 0.0014; Figure 3D), consistent with reduced locomotor and exploratory activity. No significant differences were observed in the time spent in the social zone between the two groups (Figure 3E).

### 3.4. Mirror Biting Test

**Figure 4:**
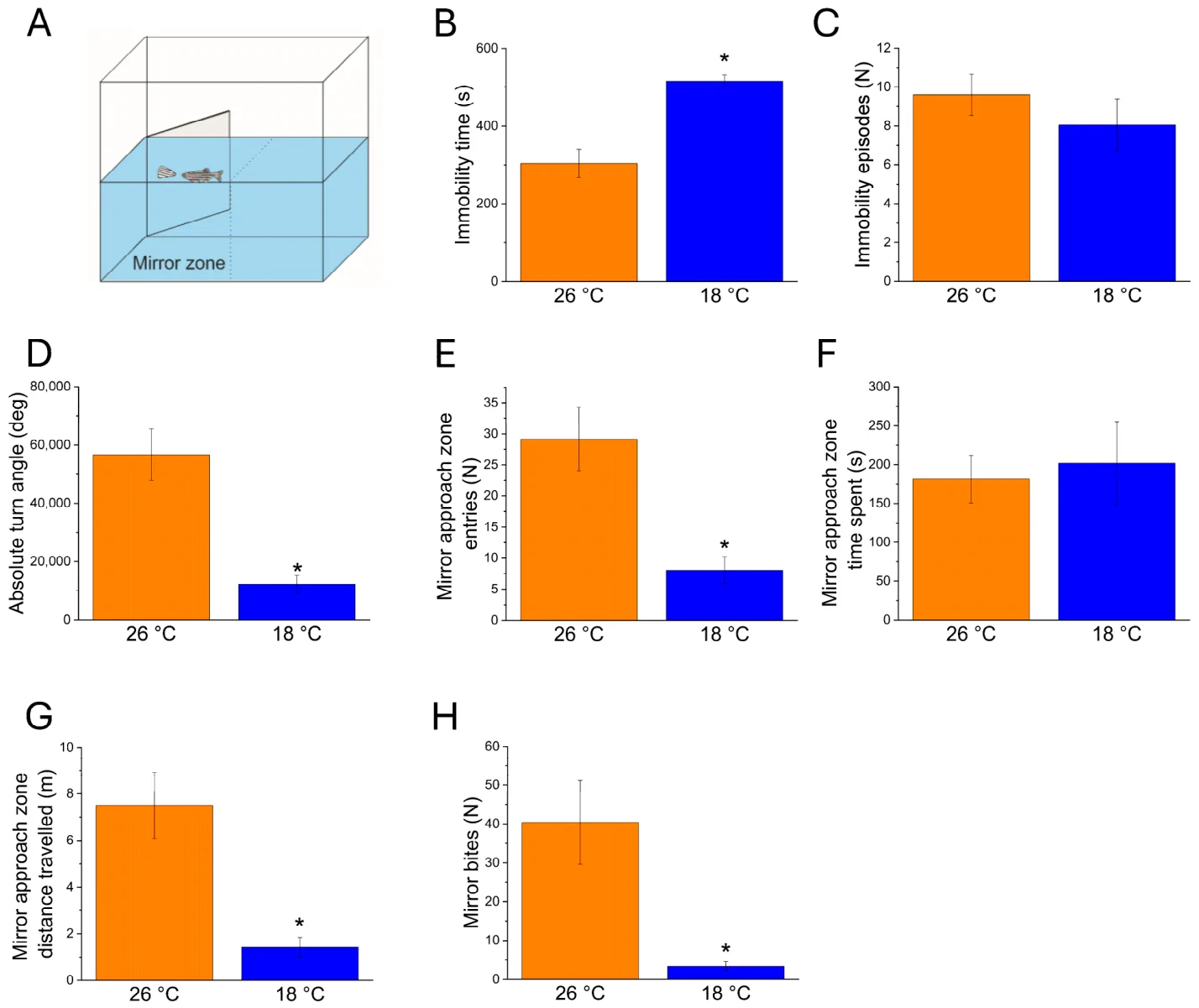
Mirror-directed behaviour following chronic exposure to low temperature. (**A**) Schematic representation of the apparatus. Whole-tank analysis: (**B**) Immobility time, (**C**) number of immobility episodes and (**D**) absolute turn angle. Mirror approach zone analysis: (**E**) Number of entries into the mirror approach zone, (**F**) time spent in the mirror approach zone, (**G**) distance travelled within the mirror approach zone and (**H**) number of mirror bites. Data are expressed as mean ± SEM. Significant differences were assessed by unpaired two-tailed *t*-tests with Welch’s correction. * *p* ≤ 0.05 versus 26 °C. *N* = 20 per group.

In the MBT (Figure 4A), zebrafish exposed to 18 °C showed a significant increase in immobility time compared with controls (t(26.03) = 5.35, *p* < 0.0001), while the number of immobility episodes did not differ significantly between groups (Figure 4B,C), further supporting a reduction in overall locomotor activity under chronic low-temperature exposure.

Fish maintained at 18 °C also exhibited a significant reduction in absolute turn angle (t(23.30) = −4.76, *p* = 0.0001), number of entries into the mirror approach zone (t(25.69) = −3.81, *p* = 0.0008), distance travelled within the mirror approach zone (t(22.35) = −4.13, *p* = 0.0004), and number of mirror bites (t(19.45) = −3.42, *p* = 0.0028) compared with fish kept at 26 °C (Figure 4D,E,G,H). By contrast, no significant difference was observed in the time spent in the mirror approach zone (Figure 4F).

## 4. Discussion

### 4.1. Overview and Study Aims

Understanding how prolonged exposure to low temperature affects behaviour is important because temperatures that are compatible with survival may nevertheless be associated with substantial changes in behavioural performance and responsiveness to environmental stimuli. In zebrafish, the behavioural consequences of acute thermal exposure and warming have been investigated more extensively than those associated with prolonged exposure to low temperature. The present study addresses this gap by providing a systematic assessment of adult zebrafish behaviour after 21 days at 18 °C, a temperature below the preferred thermal range of the species but well within its survival limits.

The study was designed to characterise the behavioural profile associated with chronic exposure to 18 °C using a battery of complementary behavioural paradigms, including the novel tank diving test (NTT), light–dark preference test (LDT), social preference test (SPT), and mirror biting test (MBT). Together, these assays provide information on locomotion, exploration, anxiety-related responses, social preference, and interactions with salient environmental stimuli.

A further objective was to compare the present findings with those previously reported following acute exposure to the same temperature for 4 days [29], in order to evaluate whether behavioural responses remained detectable after a longer exposure period.

**Figure 5:**
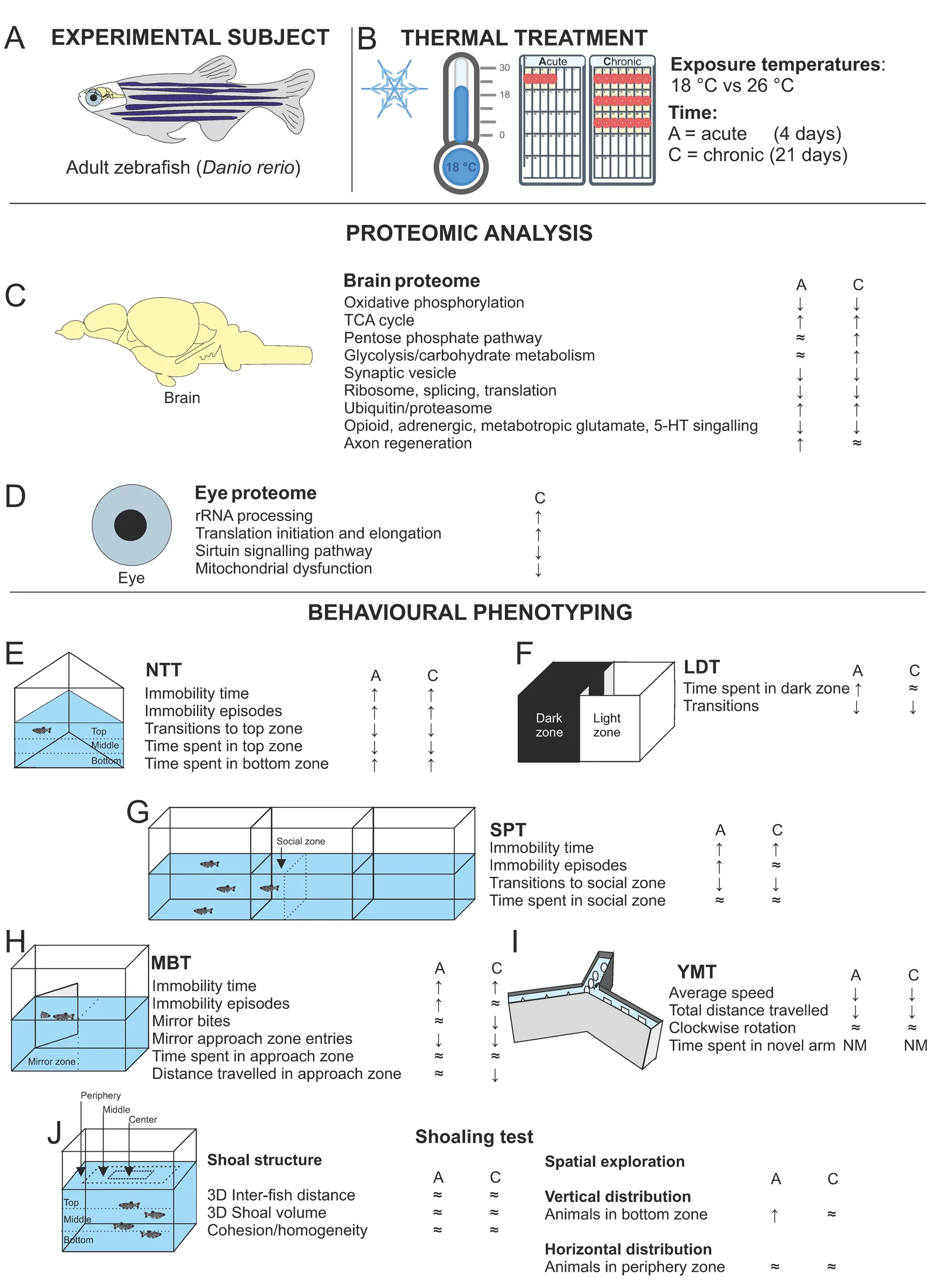
Integrated summary of molecular and behavioural alterations associated with acute and chronic exposure of adult zebrafish to low temperature. (**A**) Experimental subject: Adult zebrafish (*Danio rerio*). (**B**) Fish were exposed to 18 °C and compared with controls maintained at 26 °C under acute (4 days; (**A**)) or chronic (21 days; (**C**)) conditions. The red markings on the calendar schematically indicate the duration of the thermal treatment, corresponding to 4 days for the acute exposure and 21 days for the chronic exposure. (**C**) Previously published brain proteomic data (acute: [29]; chronic: [30]) indicate that exposure to 18 °C affects major cellular and neural pathways, with several alterations persisting from acute to chronic exposure. (**D**) Previously published eye proteomic data [32] show that chronic exposure to 18 °C also affects key pathways in the eye, suggesting that thermal effects are not restricted to the brain. (**E**–**H**) Behavioural phenotyping in individual-based assays, including the novel tank diving test (NTT), light–dark preference test (LDT), social preference test (SPT), and mirror biting test (MBT), shows that exposure to 18 °C is associated with a consistent behavioural profile characterised by reduced locomotor activity, reduced exploration, and anxiety-like features. This profile is characterised by enhanced immobility, increased bottom-dwelling, and reduced interaction with environmental stimuli, with increased scototaxis under acute exposure, and is evident under both acute [29] and chronic conditions (present study). (**I**) Previously published Y-maze data (acute: [29]; chronic: [30]) further indicate altered exploratory behaviour and lack of modulation of interest in the novel arm across repeated trials. (**J**) Previously published shoaling analysis [46] shows no major changes in core shoal structure, although acute exposure is associated with increased occupancy of the lower part of the tank. Arrows indicate significant increases (↑) or decreases (↓), and ≈ indicates no significant difference, relative to the 26 °C control condition. NM indicates that the parameter was not modulated across repeated trials. This figure integrates results from the present study together with previously published molecular and behavioural data obtained under the same thermal conditions.

The present findings were also considered in relation to previously published evidence derived from complementary behavioural paradigms, including the Y-maze [29,30] and shoaling tests [46], as well as proteomic studies of the brain and eye conducted under comparable thermal conditions [29,30,32]. These behavioural and molecular datasets were obtained in separate studies and therefore cannot be directly associated at the level of individual animals or specific behavioural endpoints. The principal behavioural findings discussed throughout this section are summarised in Table 1, which provides an overview of behavioural alterations observed in adult zebrafish exposed to 18 °C under both acute (4-day) and chronic (21-day) conditions relative to fish maintained at 26 °C. Figure 5 provides a broader integrated summary of the present findings together with previously published behavioural and molecular evidence obtained under comparable thermal conditions.

### 4.2. Reduced Exploration and Anxiety-Related Responses Under Chronic Low-Temperature Exposure

A central finding of the present study is that chronic exposure to 18 °C was associated with a persistent reduction in behavioural activity and exploration across multiple behavioural paradigms. A similar decrease in spontaneous activity has also been reported in zebrafish larvae exposed to low temperatures, suggesting that temperature-dependent suppression of behavioural activity may occur across different developmental stages [47].

Interpretation of these behavioural changes requires careful consideration because many commonly used behavioural endpoints are influenced by locomotor activity. In the present study, fish exposed to 18 °C displayed increased immobility together with reductions in several exploratory measures, including vertical transitions in the NTT, transitions between compartments in the LDT, and active responses in the MBT.

Importantly, available evidence indicates that chronic exposure to low temperature does not necessarily impair locomotor capacity itself. Little and Seebacher reported that zebrafish acclimated to 18 °C for three weeks exhibited improved sustained swimming performance, while burst performance remained unchanged [48]. Similarly, we found no reduction in maximal swimming speed following acute exposure to 18 °C [29]. Consistent with these observations, fish in the present study continued to move throughout the test arenas and performed vertical transitions, compartment crossings, and exploratory movements, although less frequently than controls.

These observations suggest that the behavioural profile observed here is unlikely to reflect a simple inability to swim. Rather, it appears more consistent with a reduction in spontaneous exploration and behavioural activation. This distinction is important because locomotor variables such as swimming speed and immobility are not always directly associated with validated measures of anxiety-related behaviour [49].

Within this framework, the NTT revealed increased immobility, reduced vertical exploration, and enhanced bottom-dwelling following chronic exposure to 18 °C. This profile resembles that previously reported after acute exposure [29] and is further supported by Liu and collaborators, who found that zebrafish exposed to 18 °C for 24 h displayed reduced locomotor activity and spent less time in the upper region of a novel tank [50]. Together, these observations suggest that reduced vertical exploration is a reproducible behavioural response to acute and prolonged low-temperature exposure. This pattern is commonly interpreted as reflecting increased anxiety-related responses together with reduced exploratory drive. However, because reductions in activity were observed across multiple behavioural paradigms, a contribution of broader behavioural suppression cannot be excluded. Indeed, reduced vertical exploration may be influenced both by anxiety-related responses and by a more general reduction in behavioural activation associated with cold exposure.

The LDT produced a similar pattern. Although fish maintained at 18 °C did not spend significantly more time in the dark compartment, they showed fewer transitions between compartments. This finding is consistent with reduced exploratory behaviour and suggests that any interpretation based solely on dark preference should be made cautiously. Moreover, the strong baseline preference for the dark compartment displayed by control fish may have limited the sensitivity of this variable through a potential ceiling effect.

The MBT further supports the presence of reduced behavioural responsiveness. Fish exposed to 18 °C displayed fewer entries into the mirror-approach zone and performed fewer mirror bites. While these findings could be interpreted as reduced aggression or reduced motivation to engage with the mirror stimulus, they are also compatible with the broader reduction in behavioural activity observed throughout the study.

Taken together, the results indicate that chronic low-temperature exposure is associated with reduced exploration and behavioural responsiveness. Although several endpoints are compatible with anxiety-related responses, their interpretation should remain cautious because multiple measures may be influenced by the general reduction in behavioural activity observed under chronic cold exposure.

### 4.3. Persistence of the Low-Temperature Behavioural Profile Despite Prolonged Exposure

A primary objective of the present study was to determine whether the behavioural profile previously observed after acute exposure to 18 °C persisted during prolonged exposure.

Comparison with previously published acute data indicates substantial qualitative consistency between the acute and chronic conditions (Table 1). Increased immobility, reduced exploratory behaviour, and altered responses across several behavioural paradigms remained evident after 21 days of exposure. Although some endpoints appeared less affected than during acute treatment, these differences were limited and did not substantially modify the overall behavioural profile.

Therefore, fish maintained at 18 °C continued to display behavioural responses that differed from those observed in fish maintained at 26 °C. The persistence of these differences suggests that behavioural adjustments occurring during the first days of exposure were largely maintained throughout the experimental period. This persistence may therefore reflect successful acclimation to low temperature rather than a failure to acclimate, although the present data do not allow the timing or mechanisms of this adjustment to be determined.

However, the present behavioural data do not establish whether this profile represents incomplete acclimation, an adaptive adjustment to low temperature, or a maladaptive response. Distinguishing among these alternatives would require additional information regarding physiological performance, energy expenditure, reproductive success, survival, and other fitness-related traits.

Consequently, the present findings are best interpreted as evidence that prolonged exposure to 18 °C is associated with a persistent behavioural phenotype that remains distinct from that observed under control thermal conditions, while the biological significance of this phenotype remains to be determined.

### 4.4. Relative Resilience of Social Motivation

In contrast to the pronounced effects observed on locomotion and exploration, the social domain appeared comparatively less affected by chronic low-temperature exposure.

In the social preference test, zebrafish maintained at 18 °C displayed increased immobility and a reduced number of transitions, while the time spent in proximity to conspecifics did not differ significantly from that of control fish. This observation suggests that attraction towards conspecifics may be relatively preserved despite the overall reduction in behavioural activity.

Nevertheless, this interpretation should be approached with caution. Because fish exposed to 18 °C also exhibited increased immobility and reduced movement between zones, the unchanged occupancy of the social area may partly reflect reduced locomotion once the social zone has been reached. Consequently, the present results do not allow a complete separation between social motivation and the influence of the broader hypolocomotor state characterising fish maintained at low temperature.

The present findings are consistent with observations previously reported under acute low-temperature exposure [29] and are further supported by shoaling studies conducted under comparable thermal conditions [46]. In those experiments, both acute and chronic exposure to 18 °C produced relatively limited effects on major structural properties of the shoal, including inter-individual distance and shoal cohesion, despite reductions in activity and vertical exploration (Figure 5). Interestingly, it has been reported that adult zebrafish exposed to 18 °C for 24 h exhibited a significant reduction in inter-fish distance, indicating increased shoal cohesion under acute cold stress [50]. Although differences in exposure duration, experimental design, and shoaling metrics preclude direct comparison with the present findings, both studies suggest that social interactions are relatively preserved under low-temperature conditions despite marked reductions in locomotor and exploratory activity.

Taken together, these findings suggest that social attraction may be more resistant to thermal perturbation than exploratory behaviour. The increased shoaling cohesion reported under acute cold exposure [50], together with the limited effects of low temperature on social preference and shoal structure observed in our studies, is consistent with the possibility that maintaining proximity to conspecifics represents an adaptive response under challenging thermal conditions. Such behaviour could provide benefits including enhanced vigilance, social buffering, or reduced energetic expenditure during group swimming. However, these interpretations remain speculative and require direct experimental investigation.

### 4.5. Evidence from Complementary Exploratory Paradigms

Additional evidence from previously published Y-maze studies conducted by our group under comparable thermal conditions [29,30] provides further context for the behavioural differences observed following both acute and chronic exposure to 18 °C. In this paradigm, zebrafish exposed to low temperature showed altered patterns of exploration of the novel arm and did not display the experience-dependent modulation typically observed across repeated trials.

These findings suggest that low temperature may influence not only general locomotor and exploratory activity, but also the way behavioural responses change with experience. A similarly cautious interpretation has been adopted in other studies of cold-exposed zebrafish. For example, Liu et al. reported that adult zebrafish exposed to 18 °C for 24 h showed reduced performance in an active avoidance task and concluded that the learning process was sensitive to environmental cold stress.

Importantly, performance in both Y-maze and active avoidance paradigms reflects the contribution of multiple interacting processes, including locomotor activity, exploratory motivation, responsiveness to novelty, stress reactivity, and learning-related mechanisms. Consequently, the available evidence is more appropriately interpreted as indicating altered experience-dependent modulation of behaviour under cold exposure rather than direct evidence of generalised cognitive impairment.

Although obtained using different behavioural paradigms, these observations are broadly consistent with the reduced exploratory activity observed in the present study across the NTT, LDT, and MBT. Considered together, the findings indicate that fish maintained at 18 °C continue to display behavioural responses distinct from those of controls after prolonged exposure. However, the relative contributions of reduced locomotion, altered exploratory motivation, and other behavioural or physiological processes cannot be fully resolved from the available behavioural data.

### 4.6. Molecular Reorganisation Parallels Persistent Behavioural Alterations

Evidence from previously published studies conducted by our group [29,30], although not part of the present dataset, provides an important framework for interpreting the relationship between molecular and behavioural responses to low temperature.

Previous proteomic analyses showed that acute exposure to 18 °C induces extensive remodelling of the brain proteome, involving pathways related to energy metabolism, synaptic function, and neurotransmission, in parallel with a behavioural profile characterised by reduced locomotion, increased immobility, and diminished exploratory activity [29]. Although causality cannot be established, the coexistence of molecular and behavioural changes suggests that both levels of biological organisation respond in a coordinated manner to thermal perturbation.

Proteomic analyses performed after chronic exposure demonstrated that several molecular alterations persist after 21 days, while additional pathways become differentially regulated during prolonged exposure [30]. These findings indicate that chronic low-temperature exposure is associated with substantial molecular reorganisation rather than a simple return to the control state. In particular, pathways involved in cellular metabolism, protein turnover, synaptic processes, and neurotransmission remained markedly affected under chronic conditions.

The present study was not designed to associate specific molecular alterations with individual behavioural endpoints, nor does it permit direct causal interpretations. Nevertheless, at a systems level, the persistence of behavioural differences alongside extensive molecular remodelling is consistent with the idea that prolonged low-temperature exposure affects the overall functional state of the nervous system.

Recent findings from our group further indicate that the effects of chronic temperature exposure are not restricted to the brain. Chronic exposure to non-optimal temperatures also alters protein expression in the eye of adult zebrafish [32], suggesting that thermal effects involve multiple neural-derived structures. This observation is particularly relevant because several behavioural paradigms employed in the present study rely, at least in part, on the processing of visual information.

Although the present data do not allow direct links between ocular proteomic alterations and specific behavioural outcomes, they suggest that behavioural responses to chronic low temperature may reflect changes occurring at multiple levels of neural organisation, including both central and peripheral sensory structures. Within this broader framework, the maintenance of behavioural differences after 21 days is consistent with persistent biological responses to low temperature, although the functional significance of these responses remains to be established.

Future studies integrating behavioural analyses with targeted molecular, physiological, and neurofunctional approaches will be required to clarify the mechanisms underlying these responses.

### 4.7. Ecological and Functional Implications

From an ecological perspective, the present findings suggest that temperatures within the survival range of zebrafish may nevertheless be associated with substantial changes in behavioural performance.

Fish maintained at 18 °C displayed reduced locomotor activity, diminished exploration, increased immobility, and reduced active engagement with environmental stimuli. Although the present study was conducted under controlled laboratory conditions, behaviours such as locomotion, exploration, and responsiveness to environmental cues are fundamental components of ecological performance and may influence the way animals interact with their surroundings.

However, the present study did not directly evaluate ecologically relevant outcomes such as foraging efficiency, predator avoidance, competitive ability, reproductive success, survival, or fitness. Consequently, the ecological consequences of the behavioural profile observed under chronic low-temperature exposure cannot be determined from the current data.

Future investigations integrating prolonged thermal exposure with ecologically oriented behavioural assays and relevant physiological endpoints will be necessary to determine whether the behavioural differences observed here translate into measurable effects on individual performance and fitness.

### 4.8. Limitations and Future Directions

Several limitations should be considered when interpreting the present findings.

First, thermal treatment was applied at the tank level, with a single home tank representing each temperature condition. Although behavioural measurements were collected at the level of individual fish, independent tank-level replication was not available, and potential tank-specific effects cannot be fully separated from temperature-related effects. Future studies should therefore include multiple independent replicate tanks per temperature condition, allowing tank-level variation to be appropriately incorporated into the experimental design and statistical analyses.

Second, sex was not recorded at the individual level and therefore potential sex-dependent responses to chronic low-temperature exposure could not be evaluated. Although fish were selected to achieve an approximately balanced sex ratio, behavioural analyses were not designed to assess sex as an experimental factor. Future studies incorporating individual sex identification and balanced experimental designs will be required to investigate possible interactions between temperature and sex.

Finally, the present study focused on behavioural endpoints and did not directly assess physiological performance, energy metabolism, reproductive output, survival, or other fitness-related variables. Consequently, the adaptive significance of the behavioural profile observed at 18 °C cannot be established from the available data alone.

Future studies incorporating independent tank-level replication will be important both to confirm the behavioural differences reported here and to clarify their underlying mechanisms and biological significance.

## 5. Conclusions

In conclusion, the present study shows that chronic exposure to 18 °C is associated with a distinct behavioural profile in adult zebrafish, characterised by increased immobility, reduced locomotor and exploratory activity, enhanced bottom-dwelling, and reduced active responses to the mirror stimulus. Several behavioural differences previously observed after acute exposure were still detectable after 21 days, indicating that the effects associated with low-temperature exposure extend beyond the acute phase and remain evident within the time window examined.

Attraction towards conspecifics appeared comparatively preserved. However, the broad reduction in locomotor activity observed across paradigms should be considered when interpreting endpoints related to social-zone occupancy, exploration, and mirror-directed responses. Reduced transitions, zone entries, and mirror-directed behaviours may reflect both reduced behavioural activation and changes in specific behavioural domains, whose relative contributions cannot be disentangled from the present data.

Previous molecular studies conducted under comparable thermal conditions provide a relevant biological context for the present findings by showing that prolonged low-temperature exposure is also associated with changes in the brain and eye. However, because molecular and behavioural measurements were obtained in separate studies, causal relationships between specific molecular changes and individual behavioural endpoints cannot be established.

Overall, the findings demonstrate that prolonged exposure to a low but non-lethal temperature is associated with persistent and multidimensional differences in zebrafish behaviour. The present behavioural data do not establish whether this profile represents incomplete acclimation, an adaptive adjustment, or a maladaptive response. Future studies incorporating independent tank-level replication, individual sex identification, and complementary physiological and ecologically relevant endpoints will be important for clarifying the mechanisms and functional significance of these behavioural responses.

## Figures and Tables

**Table 1:** Summary of behavioural alterations in adult zebrafish exposed to 18 °C under acute (4 days) and chronic (21 days) conditions, compared with fish maintained at 26 °C. Behavioural changes are reported as significant increases (↑), decreases (↓), or no difference (≈) for the comparison between 18 °C and 26 °C. The table integrates data obtained from the present study and from previously published work. Specifically, chronic data for the novel tank diving test (NTT), light–dark preference test (LDT), social preference test (SPT), and mirror biting test (MBT) derive from the present study; acute data for the NTT, LDT, SPT, MBT and Y-maze test (YMT) derive from [29]; chronic YMT data derive from [30]; and shoaling test data under both acute and chronic conditions derive from [46]. Shoaling analysis was performed on groups of four animals, whereas the other behavioural tests were individual-based assays. NM = not modulated.

		18 °C vs. 26 °C	
		Acute	Chronic
Novel tank diving test	Immobility time (s)	↑	↑
	Immobility episodes (N)	↑	↑
	Transitions to top zone (N)	↓	↓
	Time in top zone (s)	↓	↓
	Time in bottom zone	↑	↑
Light–dark preference test	Time spent in dark zone	↑	≈
	Transitions (N)	↓	↓
Social preference test	Immobility time (s)	↑	↑
	Immobility episodes (N)	↑	≈
	Time spent in social zone	≈	≈
	Transitions (N)	↓	↓
Mirror biting test	Immobility time (s)	↑	↑
	Immobility episodes (N)	↑	≈
	Mirror bites (N)	≈	↓
	Mirror approach zone: entries (N)	↓	↓
	Mirror approach zone: time spent (s)	≈	≈
	Mirror approach zone: distance travelled (m)	≈	↓
Y-maze test	Average speed (m/s)	↓	↓
	Total distance travelled (m)	↓	↓
	CW rotations	≈	≈
	Time spent in novel arm (%)	NM	NM
Shoal test	3D inter-fish distance	≈	≈
	3D shoal volume	≈	≈
	Distance to centroid	≈	≈
	Homogeneity index	≈	≈
	Animals in bottom zone	↑	≈
	Animals in middle zone	↓	≈
	Animals in top zone	≈	≈
	Animals in centre zone	≈	≈
	Animals in middle zone	≈	≈
	Animals in peripheral zone	≈	≈

## Data Availability

The raw data supporting the conclusions of this article will be made available by the authors on request.

## Supplementary Materials

The following supporting information can be downloaded at https://www.mdpi.com/article/10.3390/ani16182945/s1. Table S1. Statistical details of behavioural comparisons between adult zebrafish exposed to 18 °C for 21 days and controls maintained at 26 °C. Behavioural parameters refer to the novel tank diving test (NTT), light–dark preference test (LDT), social preference test (SPT), and mirror biting test (MBT). Results are reported for unpaired two-tailed Student’s *t*-tests assuming equal variances and for unpaired two-tailed *t*-tests with Welch’s correction. Statistical interpretation in the manuscript was based on Welch’s correction. For consistency, t values are reported according to the contrast 18–26 °C; therefore, positive t values indicate higher values in fish exposed to 18 °C, whereas negative t values indicate lower values in fish exposed to 18 °C relative to controls maintained at 26 °C. Data correspond to the behavioural analyses shown in Figure 1, Figure 2, Figure 3 and Figure 4. df, degrees of freedom; p, probability value. *N* = 20 fish per group.

## Abbreviations

3D	three-dimensional
df	degree of freedom
LDT	light–dark preference test
MBT	mirror biting test
NM	not modulated
NTT	novel tank diving test
SEM	standard error of the mean
SPT	social preference test
YMT	Y-maze test
