# Supplementary material for: Effects of Chronic Low-Temperature Exposure on the Behaviour of Adult Zebrafish (Danio rerio)
Source: Animals (Basel). 2026 Sep 19;16(18):2945. doi: 10.3390/ani16182945 (PMC13603280; doi:10.3390/ani16182945)
Supplement: Supplementary file 1 [file animals-16-02945-s001.zip › Table S1.pdf]

Table S1. Statistical details of behavioural comparisons between adult zebrafish exposed to 18 °C for 21 days and controls maintained at 26 °C. Behavioural parameters refer to the novel tank diving test (NTT), light–dark preference test (LDT), social preference test (SPT), and mirror biting test (MBT). Results are reported for unpaired two-tailed Student's t-tests assuming equal variances and for unpaired two-tailed t-tests with Welch's correction. Statistical interpretation in the manuscript was based on Welch's correction. For consistency, t values are reported according to the contrast 18 °C − 26 °C; therefore, positive t values indicate higher values in fish exposed to 18 °C, whereas negative t values indicate lower values in fish exposed to 18 °C relative to controls maintained at 26 °C. Data correspond to the behavioural analyses shown in Figures 1–4. df, degrees of freedom; p, probability value. N = 20 fish per group.

		**Student's t-test**			**Welch's correction**		
		t	df	p	t	df	p
**NTT**	Immobility time (s)	3.28	38.00	0.0022	3.28	22.42	0.0034
	Immobility episodes (N)	2.35	38.00	0.0240	2.35	37.83	0.0241
	Transitions to bottom zone (N)	-4.23	38.00	0.0001	-4.23	29.38	0.0002
	Transitions to middle zone (N)	-3.71	38.00	0.0007	-3.71	31.39	0.0008
	Transitions to top zone (N)	-2.80	38.00	0.0079	-2.80	32.56	0.0084
	Time spent in bottom zone (s)	2.59	38.00	0.0136	2.59	34.98	0.0140
	Time spent in middle zone (s)	-2.66	38.00	0.0113	-2.66	36.80	0.0114
	Time spent in top zone (s)	-2.20	38.00	0.0341	-2.20	29.81	0.0358
**LDT**	Time spent in dark zone	-0.22	38.00	0.8270	-0.22	36.27	0.8271
	Transitions (N)	-2.20	38.00	0.0339	-2.20	30.85	0.0354
**SPT**	Immobility time (s)	2.24	38.00	0.0311	2.24	26.46	0.0338
	Immobility episodes (N)	1.51	38.00	0.1406	1.51	37.18	0.1408
	Time spent in the social zone	-0.08	38.00	0.9364	-0.08	27.05	0.9365
	Transitions (N)	-3.44	38.00	0.0014	-3.44	37.35	0.0014
**MBT**	Immobility time (s)	5.35	38.00	<0.0001	5.35	26.03	<0.0001
	Immobility episodes (N)	-0.91	38.00	0.3680	-0.91	36.13	0.3683
	Absolute turn angle (deg)	-4.76	38.00	<0.0001	-4.76	23.30	0.0001
	Mirror approach zone: entries (N)	-3.81	38.00	0.0005	-3.81	25.69	0.0008
	Mirror approach zone: time spent (s)	0.33	38.00	0.7420	0.33	30.29	0.7425
	Mirror approach zone: distance travelled (m)	-4.13	38.00	0.0002	-4.13	22.35	0.0004
	Mirror bites (N)	-3.42	38.00	0.0015	-3.42	19.45	0.0028
